# 101 The Burden of Burns: An Analysis of Public Health Measures

**DOI:** 10.1093/jbcr/irae036.100

**Published:** 2024-04-17

**Authors:** Anastasiya Ivanko, Anna Garbuzov, Jonathan E Schoen, Randy D Kearns, Denise Danos, Erica Murata, Bart D Phillips, Herb A Phelan, Jeffrey E Carter

**Affiliations:** University Medical Center (LSU Health), New Orleans, LA; Louisiana State University, New Orleans, Louisiana; University of New Orleans, New Orleans, LA; LSUHSC New Orleans, New Orleans, LA; BData, Inc., Chicago, IL; BData, Inc, Edina, MN; Louisiana State University Health Sciences Center- New Orleans, New Orleans, LA; University Medical Center (LSU Health), New Orleans, LA; Louisiana State University, New Orleans, Louisiana; University of New Orleans, New Orleans, LA; LSUHSC New Orleans, New Orleans, LA; BData, Inc., Chicago, IL; BData, Inc, Edina, MN; Louisiana State University Health Sciences Center- New Orleans, New Orleans, LA; University Medical Center (LSU Health), New Orleans, LA; Louisiana State University, New Orleans, Louisiana; University of New Orleans, New Orleans, LA; LSUHSC New Orleans, New Orleans, LA; BData, Inc., Chicago, IL; BData, Inc, Edina, MN; Louisiana State University Health Sciences Center- New Orleans, New Orleans, LA; University Medical Center (LSU Health), New Orleans, LA; Louisiana State University, New Orleans, Louisiana; University of New Orleans, New Orleans, LA; LSUHSC New Orleans, New Orleans, LA; BData, Inc., Chicago, IL; BData, Inc, Edina, MN; Louisiana State University Health Sciences Center- New Orleans, New Orleans, LA; University Medical Center (LSU Health), New Orleans, LA; Louisiana State University, New Orleans, Louisiana; University of New Orleans, New Orleans, LA; LSUHSC New Orleans, New Orleans, LA; BData, Inc., Chicago, IL; BData, Inc, Edina, MN; Louisiana State University Health Sciences Center- New Orleans, New Orleans, LA; University Medical Center (LSU Health), New Orleans, LA; Louisiana State University, New Orleans, Louisiana; University of New Orleans, New Orleans, LA; LSUHSC New Orleans, New Orleans, LA; BData, Inc., Chicago, IL; BData, Inc, Edina, MN; Louisiana State University Health Sciences Center- New Orleans, New Orleans, LA; University Medical Center (LSU Health), New Orleans, LA; Louisiana State University, New Orleans, Louisiana; University of New Orleans, New Orleans, LA; LSUHSC New Orleans, New Orleans, LA; BData, Inc., Chicago, IL; BData, Inc, Edina, MN; Louisiana State University Health Sciences Center- New Orleans, New Orleans, LA; University Medical Center (LSU Health), New Orleans, LA; Louisiana State University, New Orleans, Louisiana; University of New Orleans, New Orleans, LA; LSUHSC New Orleans, New Orleans, LA; BData, Inc., Chicago, IL; BData, Inc, Edina, MN; Louisiana State University Health Sciences Center- New Orleans, New Orleans, LA; University Medical Center (LSU Health), New Orleans, LA; Louisiana State University, New Orleans, Louisiana; University of New Orleans, New Orleans, LA; LSUHSC New Orleans, New Orleans, LA; BData, Inc., Chicago, IL; BData, Inc, Edina, MN; Louisiana State University Health Sciences Center- New Orleans, New Orleans, LA

## Abstract

**Introduction:**

Accurate analysis of injuries and illnesses is paramount when allocating resources for education, prevention, recovery, and research. Inconsistencies in public health reports and increased lobbying/marketing by medical nonprofits has obscured the perception of burn injury (BI) leading to inequities in healthcare access and funding of reconstruction and research. A primary example of these developments is the frequently referenced and inaccurate BI statistics. Our study aims to compare national references reporting the incidence of BI and the related sequela in the U.S.

**Methods:**

The American Burn Association Burn (ABA) Injury Summary Report (BISR), ABA Fact Sheet, Centers for Disease Control and Prevention (CDC) Web-based Injury Statistics Query and Reporting (WISQARS) database, the CDC National Center for Health Statistics’ National Hospital Ambulatory Medical Care Survey (NHAMCS), and the commercially available claims databases were queried for 2020 burn admissions and emergency department (ED) visits. Non-U.S. reports, claims, or analyses were excluded from our review. The costs of BI were reported in U.S. dollars where available. Claims analyses were conducted using ICD-10 BI codes coupled with burn DRG for admission, and CPT for surgical intervention from a commercially available database incorporating managed care, Medicare, and Medicaid payors.

**Results:**

The ABA Fact Sheet reported 486,000 BI annually. The 2021 ABA BISR data represented burn admissions at approximately 100 burn centers (BC) excluding non-BC, ED, and BC not participating in BCQP. CDC’s WISQARS database reported 3,529 burn-related deaths resulting in 45,135 years of potential years of life lost, 287,926 non-fatal BI from fire/flame and contact with 76.1% of patients treated in ED only and total costs of $42.4B. Mechanisms such as scald, chemical, electrical, radiation, and inhalation injury were not included. The CDC NHAMCS reported 359,000 BI presenting to ED (23% increase from CDC WISQARS). Our analysis from claims databases demonstrated over 698,555 BI as the primary code likely underreporting self-pay/uninsured, military facilities, and endowment or community-based hospitals not submitting claims data.

**Conclusions:**

Our study demonstrates a large variability in the incidence of BI. Given the increased population noted in the U.S. 2020 census, the true number of BI is likely closer to the claims database suggesting a substantial misunderstanding in the burden of burns. We additionally noted no ICD-10 codes to characterize the sequela of BI limiting our assessment of post-acute burn care challenges. Future efforts to improve CDC reporting policies and new ICD-10 codes capturing BI sequela are essential to improve advocacy and understanding of our patients and their healthcare needs.

**Applicability of Research to Practice:**

This abstract will help clinicians, patients, and investigators better advocate for population health research in burn injury.

